# Sob o solo pestilencial da mentira: informações falsas e negacionismo na epidemia de varíola no Ceará, 1900-1905, e na pandemia de covid-19 no Brasil, 2020-2022

**DOI:** 10.1590/S0104-59702024000100061

**Published:** 2024-12-16

**Authors:** Érica Cavalcante Lima, Zilda Maria Menezes Lima

**Affiliations:** i Doutora, Programa de Pós-graduação em Educação/Universidade Federal do Ceará; assistente em administração, Pró-reitoria de Gestão de Pessoas/Universidade Federal do Ceará. Fortaleza – CE – Brasil ericalimaufc@bol.com.br; ii Professora, Curso de História/Universidade Estadual do Ceará. Fortaleza – CE – Brasil zilda.lima@uece.br

**Keywords:** Vacinação, Varíola, Covid-19, Informações falsas, Negacionismo, Vaccination, Smallpox, Covid-19, Misinformation, Denialism

## Abstract

O artigo traça um paralelo entre dois fenômenos patológicos de grande impacto social em contextos diversos: a epidemia de varíola ocorrida no Ceará, entre 1900 e 1905, e a pandemia de covid-19 no Brasil, entre 2020 e 2022. Apesar da distância histórico-temporal entre eles, os eventos guardam importantes semelhanças nas formas como foram conduzidos politicamente, sobretudo no que diz respeito à utilização de notícias e informações falsas quanto à emergência de implementar a vacinação em massa. Nesse diálogo, fizemos amplo uso de fontes hemerográficas nas considerações em torno da covid-19, bem como dos escritos do farmacêutico Rodolfo Teófilo, principal personagem na campanha de vacinação contra a varíola no Ceará.

## Abstract (en)

The article draws a parallel between two pathological phenomena with great social impact in different settings: the smallpox epidemic in Ceará, northeastern Brazil, between 1900 and 1905, and the covid-19 pandemic in Brazil between 2020 and 2022. Despite the historical and temporal distance between these two episodes, there are some significant similarities in the ways they were conducted politically, especially with regard to the use of fake news and misinformation on the emergency of implementing mass vaccination. The analysis makes extensive use of press reports concerning the covid-19 pandemic and writings by the pharmacist Rodolfo Teófilo, a key figure in the smallpox vaccination campaign in Ceará.

A longa história do combate às epidemias, pelo menos desde as últimas décadas do século XIX, quando o Estado começou a agir com mais ênfase e de modo mais sistemático em relação ao que se entendia como saúde pública, tem mostrado que a visão e a maneira de agir dos governantes são cruciais. Não somente para determinar os investimentos e as ações a executar no combate às enfermidades e em sua erradicação, mas também para legislar sobre a vida e a morte dos sujeitos a partir de seus instrumentos de intervenção política.

Temos clareza de que tais acontecimentos mórbidos não se restringem ao dado biológico e estatístico do adoecimento, da cura ou da morte dos sujeitos, mas são repletos de desdobramentos que falam dos corpos acometidos (ou não) por uma enfermidade, curados (ou não), poupados (ou não) da morte. Aspectos que, nas sociedades modernas, estão profundamente conectados ao papel desempenhado por seus governantes, sobretudo pelo fato de as epidemias recaírem mais fortemente sobre indivíduos em situações de aprofundada exclusão, pobreza e exploração, em tese, os que mais dependem do Estado.

Diante dessas questões, este artigo traça um paralelo entre dois fenômenos patológicos de grande impacto social vivenciados no Brasil em contextos bastante diferentes, mas que, apesar da considerável distância histórico-temporal entre si, guardam importantes semelhanças nas formas como foram conduzidos politicamente por seus governantes, sobretudo no que diz respeito à principal ferramenta de combate a essas patologias em ambas as ocasiões: a vacinação. Referimo-nos à epidemia de varíola no Ceará, entre 1900 e 1905, e à pandemia de covid-19 no Brasil, entre 2020 e 2022, que, dois anos após sua eclosão, conseguiu ser controlada com a implementação de um plano emergencial de vacinação em massa.

O objetivo deste texto centra-se na explanação e análise desses dois eventos significativos para a história da saúde pública do Brasil, quanto às aproximações nas formas com que foram geridos pelos principais representantes do Estado no momento da crise: ambos expressaram-se em posicionamentos omissos, refletidos em ausência ou insuficiência de medidas de contenção das doenças; negligência diante da necessidade de promover e de fazer cumprir protocolos de segurança; tentativa de eximir-se da responsabilidade de prestar assistência efetiva aos atingidos; difusão de declarações negacionistas diante das evidências que demonstravam o alastramento e a gravidade das enfermidades; e, sobretudo, ferrenha oposição à principal forma de controle da proliferação das patologias em questão, a vacina, ainda que sob argumentações diferentes.

A elaboração de um estudo que se debruce sobre as deliberações políticas na gestão das duas epidemias mencionadas não se pode furtar a apontar os supostos antagonismos entre a defesa da vida e a defesa de interesses de setores específicos de uma dada coletividade, posto ressaltar que os eventos epidêmicos envolvem interesses múltiplos de variados grupos que reagem e interagem com as enfermidades segundo perspectivas diferentes (Nascimento, Silveira, 2018). Muitas vezes, as ideias que moldam os alicerces das sociedades – que, no tocante às capitalistas, como a brasileira, consistem na economia – não são aquelas que incluem a maioria da população e, por trás de discursos supostamente progressistas, mal escondem uma perspectiva desalentadora e desumana sobre a vida dos sujeitos.

Nesse sentido, ao acionar processos históricos do passado, tal como a epidemia de varíola no Ceará, ocorrida há cerca de 120 anos, e a pandemia de covid-19, controlada em 2022, após dois anos de forte crise sanitária mundial, mais válido do que julgar os comportamentos dos personagens de outras épocas e contextos, é sugerir uma aproximação em eventos apartados por mais de um século, mas que guardam desconcertantes similitudes.

Ressaltamos que para a compreensão, no presente, das realidades que estamos a vivenciar, como bem destaca Butler (2017), acionar processos históricos do passado nos possibilita ampliar nossa capacidade de reflexão, pois nos permite enxergar os fatos não apenas com o olhar emocionado e confuso de quem os vivencia no “calor da hora”, mas tendo uma perspectiva exógena do que experienciamos. A leitura de eventos semelhantes experimentados por outros sujeitos, em outras épocas, viabiliza nos abrir ao passado, colocar-nos, quem sabe, a serviço de outro devir.

Dessa maneira, entendemos que traçar paralelos entre fenômenos epidêmicos de grande impacto, como os que ora elencamos, pertencentes a períodos históricos diferentes, ajuda-nos a tecer leituras mais problematizadoras dos eventos, a ampliar nossa percepção para realidades e temporalidades que existem e coexistem em cada momento da história, a não repetir dicotomias, a formular outros esquemas de pensamento. Em outras palavras, ajuda-nos a sair do nosso lugar, para enxergar o que, imersos apenas em nosso meio, dificilmente conseguiríamos ver.

Para a elaboração deste estudo, no que diz respeito à epidemia de varíola no Ceará, utilizamos, de modo preponderante, o livro *Varíola e vacinação no Ceará*, de autoria do farmacêutico, escritor e intelectual baiano, radicado naquele estado, Rodolfo Marcos Teófilo (1853-1932).^1^ Esse livro, dividido em dois volumes, é obra de gênero híbrido, visto que não se restringe ao puro relato científico de como se dera seu trabalho na campanha de vacinação contra a varíola no Ceará. A obra foi escrita em primeira pessoa, em que o cientista é, também, narrador e testemunha ocular dos desdobramentos decorrentes do fenômeno patológico que descreve como protagonista.

Todo o registro realizado por Teófilo visava, principalmente, justificar a razão de sua incansável campanha de vacinação, atrelada a sua iniciativa individual e particular de fabricar a vacina e de aplicá-la em todos os cidadãos cearenses, que, no início do século XX, voltavam a ser devastados pela epidemia de varíola, a qual, cerca de 12 anos antes (1877-1878), havia ceifado mais de 27 mil vidas em apenas dois meses, na cidade de Fortaleza com pouco mais de cem mil habitantes (Pinheiro, 2017).

No primeiro volume dessa obra (Teófilo, 1910), o autor, além de relatar todo o processo de fabricação da vacina, bem como da campanha vacinal junto à população por ele empreendida, a partir de 1900, descreve a situação calamitosa em que se encontrava a população cearense, sobretudo os retirantes, principais acometidos pelo flagelo, que, “fugidos” da chamada “seca dos três setes” (1877-1878-1879), viviam em condições extremamente precárias nos abarracamentos localizados nas periferias de Fortaleza, destinados a seu isolamento. Relaciona, ainda, as ações em defesa da saúde pública não implementadas pelo governo da época.

No segundo volume, o autor descreve – e comprova, por meio de grande quantidade de documentos, entre os quais os relatórios da Inspetoria de Higiene do Ceará, mensagens dos presidentes do estado e cartas, além de inúmeras publicações de jornais da época, incluindo periódicos de outros estados do país (*Diário de Pernambuco*, PE; *Diário da Bahia*, BA; *O País*, RJ; *A Folha Nova*, RJ) – a hostilidade com que o governo do estado, representado pela oligarquia acciolyna,^2^ infligiu a sua campanha de vacinação em massa da população cearense.

Por meio de calúnias à pessoa de Rodolfo Teófilo e da manipulação e divulgação de notícias falsas quanto à eficácia e à segurança da vacina produzida e distribuída gratuitamente pelo farmacêutico, representantes do governo do estado declararam abertamente oposição a seu trabalho. Tinham conhecimento de que a vacina consistia na principal ferramenta de combate à doença, pois, desde os anos finais do século XIX, era fabricada e distribuída pelo Instituto Soroterápico Federal, tornando-se, no Brasil, obrigatória por lei em 1904, diante da acentuada queda no número de acometidos após o processo de vacinação (Lira Neto, 1999).

O próprio governo da província do Ceará, quando do alastramento da epidemia de varíola em 1878-1879, havia tentado empreender a vacinação contra a doença. A medida, todavia, não surtiu bons resultados em virtude da qualidade da linfa enviada pela capital federal, que, por causa da demora no transporte, chegava ao Ceará comprometida em sua eficácia. Por esse motivo, Teófilo insistia na urgência de se criar um instituto vacinogênico local (Lira Neto, 1999).

Apesar de todas as provas rigorosamente publicadas por Teófilo em jornal oficial, que atestavam a qualidade da linfa por ele produzida, como, por exemplo, o certificado de qualidade expedido pelo Instituto de Patologia Experimental de Manguinhos, no Rio de Janeiro, hoje Instituto Oswaldo Cruz (IOC),^3^ bem como pelas estatísticas, que demonstravam as drásticas quedas no número de acometidos pela varíola ano a ano, após a implementação da vacinação em massa, o governo do estado insistia em não incentivar o trabalho pioneiro de Teófilo. Ao contrário, contribuiu para a desinformação da população, que se recusava a ser vacinada por influência dos rumores de que as pessoas morreriam ou seriam acometidas por outras enfermidades, caso fossem inoculadas pela vacina do farmacêutico. Segundo Teófilo (1910), esse era o obstáculo mais difícil de transpor no processo de vacinação.

Nesse sentido, a obra citada lança luz sobre o fato de que, na gestão política de fenômenos patológicos de grande impacto, os governos nem sempre voltam suas atenções para a elaboração de estratégias que possam dirimir o avanço de eventos que ceifam, de maneira cruel, a vida de um enorme contingente de indivíduos. É o que destaca Benchimol (2001) ao analisar a condução da epidemia de febre amarela no Rio de Janeiro, por parte do governo imperial, em 1849-1850, que, ao recusar omitir-se diante de tamanho flagelo social, encontrou grande resistência por parte considerável dos parlamentares, que achava desnecessária a disponibilização de verbas para socorros públicos, bem como implementação de quarentena nos portos, por considerar as medidas descabidas e extremamente prejudiciais à economia do país. Para esse autor, as discordâncias na gestão das epidemias nunca se restringem a questões meramente sanitárias, ao contrário, evidenciam os interesses políticos, culturais e econômicos que dão corpo e voz a tais dissonâncias (Benchimol, 2001).

Não fosse a experiência que vivenciamos nos últimos dois anos, 2020-2022, com a pandemia de covid-19, a qual, apenas no Brasil, levou a óbito quase setecentas mil pessoas, poderíamos pensar que a oposição do governo do Ceará à implementação da campanha vacinal promovida por Rodolfo Teófilo contra a varíola, no início do século XX, fosse fruto do desconhecimento acerca dos benefícios da vacina, da pouca experiência com sistematização de políticas/ações de saúde pública ou mesmo da pouca crença na ciência. Embora já ocupasse algum espaço na condução das políticas públicas empreendidas pela capital federal da República, a ciência ainda não gozava do *status* e da credibilidade que, ao longo do século XX, sobremaneira, conseguiu alcançar, principalmente no Ceará.

Mais de cem anos após a luta empreendida por Rodolfo Teófilo em defesa da vacinação como a principal forma de enfrentamento a uma doença infectocontagiosa, constatamos, todavia, que o governo brasileiro, representado, predominantemente, na figura do então presidente da República Jair Messias Bolsonaro (2019-2022), tornou-se declarado opositor à vacinação em massa contra a covid-19. Mesmo diante de benefícios incontestáveis à saúde pública promovidos pelas vacinas, aproveitou-se de argumentos que não demonstravam qualquer compromisso com a verdade e não apresentavam nenhum tipo de fundamentação científica confiável (Amado, 2022).

No que diz respeito à análise da gestão da pandemia de covid-19 promovida pelo maior representante político do povo brasileiro, o presidente da República, utilizaremos como fontes as resoluções, instruções normativas, leis, portarias e medidas provisórias do governo federal, além de vetos e decretos presidenciais, indicadores da postura autoritária, negacionista e obscurantista do presidente, que percebia como opositor qualquer gestor promovendo ações visando ao cumprimento das determinações da Organização Mundial de Saúde (OMS) acerca dos protocolos de segurança, que, num primeiro momento, previa como medida primordial o isolamento social.

Tal aspecto trazia inúmeros desafios ao pleno funcionamento da economia, setor que, conforme as inúmeras declarações do presidente, “não poderia parar”, ainda que várias autoridades sanitárias, entre as quais dois ministros da Saúde, médicos, Luiz Henrique Mandetta e Nelson Teich, indicados em seu governo, reforçassem continuamente os riscos que incorriam sobre a população caso a medida do isolamento não fosse plenamente efetivada (Gazeta do Povo, 15 maio 2020).

As falas públicas do ex-presidente, veiculadas via redes sociais (Facebook, Twitter, Instagram e WhatsApp), também se revelaram importantes fontes de pesquisa para este estudo, tendo em vista que esses veículos foram amplamente utilizados como estratégia de aproximação com seu eleitorado e, conforme o discurso, consistiriam, durante seu governo, em verdadeiros “registros oficiais” de suas opiniões sobre a covid-19 e das medidas a ser implementadas em seu combate.

Tais registros evidenciaram a desimportância social que conferiu à doença, o modo como percebia as eventuais mortes que dela pudessem decorrer e, sobretudo, como lidou com o surgimento da principal ferramenta para o controle real da proliferação da doença, a vacina, deixando claras suas linhas de ação na gestão da pandemia, quais sejam: relaxamento das medidas de isolamento em prol da economia; incentivo ao uso de medicamentos sem comprovação científica como alternativa de “tratamento precoce” da enfermidade; e vacinação não compulsória, como suposta defesa da liberdade individual. São todas estratégias claramente baseadas em lógica populista e neoliberal que delega sua responsabilidade como gestor de uma crise sanitária à decisão particular de cada cidadão, merecedor, conforme sua narrativa, do direito de escolher como lutar pela própria vida (Monari et al., 2021).

Paul Preciado (20 mar. 2020) destaca que, sob o prisma oferecido por Michel Foucault (2012), Roberto Espósito (2010) e Emily Martin (1994), ao observar como uma comunidade estrutura a própria soberania política, temos melhor compreensão acerca das formas e proporções que tomarão suas epidemias e como serão enfrentadas, e também de que modo os distintos fenômenos patológicos de grande impacto materializam na esfera dos corpos individuais as obsessões que dominam a gestão política da vida e da morte dos sujeitos em um período determinado. Corroborando essa compreensão, no tópico seguinte, buscaremos demonstrar, a partir do paralelo entre a gestão política dos dois eventos em questão, de que forma o negacionismo e a tática de proliferação de notícias falsas, ainda que em diferentes medidas e por diferentes razões – próprias de suas épocas – representaram, além de um reflexo obscurantista de seus gestores, uma estratégia política.

## O negacionismo como perversa estratégia política

Apesar de estarmos vivenciando um momento conhecido como “pós-verdade”, que, conforme destaca El-Jaick (2019), se caracteriza por uma espécie de “cinismo contemporâneo”, que banaliza a estratégia cética de suspender o juízo sobre qualquer declaração assertiva e, deliberadamente, descredibiliza a “verdade dos fatos”, mesmo depois de confirmados (posteriormente) por fontes confiáveis, o fenômeno do negacionismo, convenientemente aliado à estratégia de proliferação de informações/notícias falsas, não é novidade para o campo da história da saúde e das doenças.

Tal estratégia, longe de querer incentivar a criticidade na população quanto à absorção de informações inverídicas, tem demonstrado intenções contrárias. O estímulo ao “cinismo”, que busca incutir a compreensão de que tudo o que se sabe, até por parte da ciência, é fruto de mera opinião, não reflete ingenuidade e, menos ainda, decorre de alguma teoria epistemológica que reconhece a falibilidade dos argumentos científicos para os fatos, os de ordem fisiopatológica incluídos. A dúvida, o descrédito e a deslegitimação das afirmações científicas que contrariam interesses econômicos, políticos e culturais têm sido promovidos, em diferentes contextos, como uma importante estratégia de perpetuação de poder. A difusão da ignorância, nesse sentido, torna-se estratégia pública e *commodity* preciosa com vultosos investimentos (Latour, 2018).

Ao falar a respeito do fenômeno do negacionismo, sobretudo aquele que se coloca antagonicamente às verdades científicas, é importante destacar que não nos referimos a divergências de interpretações ou pontos de vista construídos a partir da experiência vivida, mas daquele que é fruto da fabulação de verdades desejosas de legitimar perante a coletividade posturas, comportamentos e atitudes que não encontram respaldo dentro do conhecido como sistemas de peritos, nos quais se incluem a ciência, a estatística e a esfera pública, estruturas que sustentaram o arranjo social da modernidade durante boa parte do século XX (Cesarino, 2021a).

Tratamos, na verdade, do negacionismo construído a partir da busca por explicações a demandas pessoais pautadas apenas no conforto de suas expectativas, desprendidas da obrigatoriedade de comparação com qualquer parâmetro de realidade, favorecendo um relativismo niilista que transforma fatos em opiniões, como se fosse suficiente evocar repetidamente uma afirmação para lhe conferir o *status* de “verdade”. Modificando o regime de verdade baseado nas instituições para o consolidar nas emoções, nas crenças e nas experiências, negando fatos objetivos para se apoiar em “fatos alternativos”.

Dessa forma, quando um governo opta pela veiculação de verdades manipuladas, escolhendo difundir determinadas informações que, retiradas de certos contextos ou a eles acrescidas, podem produzir, coletivamente, compreensões muito distantes da realidade dos fatos, o negacionismo mostra-se excelente estratégia de dominação irrestrita. Sobretudo quando essa manipulação artificial se realiza com a cumplicidade de setores da sociedade que, claramente, se beneficiam de seus resultados. Munidos dessa compreensão, voltemos à análise dos casos concretos em que representantes dos poderes públicos utilizaram, perversamente, o negacionismo como arma política, a despeito de todo o mal que poderiam causar à população nos contextos dessas crises sanitárias, tendo em vista, como bem apontam Porto e Ponte (2003), que a transmissão de informação verídica é vital para a gestão da saúde.

## A cruzada da vacinação contra a varíola no Ceará

No tocante ao Ceará e, mais especificamente, sua capital, Fortaleza, no início do século XX, período em que acontece a epidemia de varíola que ora analisamos, sua história já vinha marcada pela ocorrência de numerosos fenômenos mórbidos, agravados, em alguma medida, pelas estiagens, mas também pelas quadras chuvosas comuns nos meses de março, abril e maio, associados a problemas de infraestrutura, que se refletiam na falta de higiene e salubridade das ruas e casas, bem como no consumo de água e alimentos contaminados. Foram muitas as pestes que assolaram o estado, das quais temos, por exemplo, registros da epidemia de febre amarela (1851-1853), de cólera (1862-1863) e de varíola (1825, 1845, 1877, 1900) (Lira Neto, 1999; Lemos, 2016).

Mesmo com todo esse histórico de fenômenos patológicos que se entrelaçam à história do estado, os governos pareciam pouco aprender com os ensinamentos deixados por esses eventos, pois, um após o outro, não buscavam, efetivamente, munir a saúde pública com aparatos profiláticos contra as doenças. O *modus operandi* das gestões para o enfrentamento das doenças resumia-se a agir após o alastramento das epidemias, quando pouco havia a fazer. Só então o governo apropriava-se, ainda de maneira insatisfatória, da condução de medidas de enfrentamento. Medidas que, muitas vezes, não tinham nenhum tipo de eficácia. É o que observamos no trecho a seguir, em relação à epidemia de varíola de 1877-1878:

À noite acendiam-se em todas as ruas vasos com alcatrão para que o fumo do piche desinfectasse a atmosfera viciada pelos micróbios da peste. Este singular modo de desinfecção foi ordenado pela ingênua Câmara Municipal, que pensava por este modo sanear a cidade. Os poderes públicos só podiam ter sufocado a epidemia se dispusessem de um instituto vacinogênico onde fosse preparada a vacina animal. Assim em poucos dias seria vacinada e revacinada toda a população de Fortaleza (Teófilo, 1910, p.18-19).

É importante destacar que, conforme salienta Gadelha (2012), até a segunda metade do século XIX, muito do que se entendia por serviços de saúde pública no Ceará consistia na contratação, por parte da Câmara Municipal, de um médico da pobreza, responsável imediato pela saúde de toda a população, o qual acumulava as funções de fiscalizar, inspecionar e atuar na Clínica da Pobreza. Somente nos momentos de agravamento da crise contínua da saúde pública, tais como nos surtos epidêmicos – bastante recorrentes – é que eram criados distritos sanitários e enfermarias provisórias.

A despeito da precariedade nas formas de enfrentar os fenômenos patológicos na cidade, sobretudo a partir da gestão da oligarquia acciolyna (1896-1912), Fortaleza viveu importante processo de modernização, remodelação e aformoseamento. Construção de praças, jardins e espaços de convivências, tais como o Passeio Público e cafés à moda parisiense; ampliação de vias urbanas; construção do mercado de ferro e do imponente Teatro José de Alencar, entre outras obras, representaram marca significativa da gestão acciolyna, que objetivava legar a seu governo os anseios dominantes da recente República, no sentido de alinhar o país ao progresso e à modernidade. Por meio de obras vistosas, que aparelhavam as elites com equipamentos modernos de sociabilidade, revestia-se a cidade com um verniz de progresso. Suas mazelas, no entanto, continuavam a avolumar-se, pois para resolver os problemas relacionados à salubridade, às secas e à pobreza pouco se fazia (Ponte, 2014).

É nesse contexto de remodelação urbana da cidade que se insere o “grito de alerta” que Rodolfo Teófilo buscou fazer ouvir. Trazendo à tona as contradições existentes numa sociedade que, almejando transformar-se, desenvolver-se, embelezar-se à moda das grandes civilizações europeias, fechava os olhos para o descaso com a saúde pública, com a falta de higiene e de profilaxia na cidade, bem como de assistência à população desvalida.

Não conhecia os subúrbios de Fortaleza. … onde está reunida a escória da população da capital cearense. Ali, a miséria e o vício se aliaram. É um arraial composto em sua maioria de mendigos, cães e urubus. … Quem já teve a ocasião de saltar em nosso porto e percorrer aquele caminho terá grande surpresa ao encontrar no centro da cidade tão belas praças ajardinadas. Ninguém dirá, subindo aquela rampa, ladeada de lama, dentro da qual desembocam os canos de esgoto da cadeia pública e do hospital da Santa Casa de Misericórdia, tendo em frente montes de lixo de altura descomunal, que vai entrar em uma cidade bastante bela, de ruas espaçosas, inundadas de luz e bafejadas por uma brisa fresca e constante (Teófilo, 1910, p.113-114).

No contexto das últimas décadas do século XIX, o inverno de 1880 parecia dar cabo dos sofrimentos do povo cearense, que, pouco tempo antes, tinha vivido os horrores da seca de 1877-1879 e da grande epidemia de varíola, dando a todos a impressão de que era chegado o tempo de paz e prosperidade. Agarrados a essa esperança, as pessoas e, pior, o governo muito cedo pareceram esquecer os lutuosos dias de outrora, pois não buscaram meios de prevenir-se daqueles flagelos, fosse por meio de obras de armazenamento de água pluvial ou por meio da implementação de um programa de vacinação no estado, sobretudo dos novos rebentos do Ceará (Sombra, 1997).

Como destaca Teófilo (1910), bem curta foi a duração de tal quimera. A seca voltou em 1888, e, com ela, a varíola. No entanto, diferente do evento ocorrido uma década antes, os estragos foram menores, porque poucos eram também os indivíduos que estavam em condições de ser atacados, visto que a população adventícia de Fortaleza era pequena (cerca de vinte mil retirantes), se comparada à de 1877, que chegou a mais de cem mil pessoas, e muitos eram sobreviventes da última grande epidemia e, por essa razão, imunes. Assim, nessa ocasião, a varíola não surtiu efeitos sociais tão danosos como em sua derradeira passagem pelo estado. Entretanto, como em 1878, ela não desapareceu completamente, fixou-se de forma endêmica na capital, onde, vez ou outra, fazia vítimas (Studart, 1910).

Pareciam poucos os ensinamentos deixados pela última grande epidemia no estado, pois todos pareciam ter esquecido como começavam e, pior, como terminavam os grandes eventos epidêmicos em que os governantes pouco se mobilizam para conter os avanços da doença que, em 1900, voltava a acometer os cearenses. As preocupações políticas estavam muito mais voltadas para o embelezamento da capital, que buscava aformosear-se, ganhar ares de civilização (Teófilo, 1910).

É interessante ressaltar que o discurso de Teófilo se insere em contexto bastante efervescente no tocante ao debate nacional de novas ideias políticas e culturais. Tais debates eram protagonizados, em grande medida, por uma classe de intelectuais que buscava fortalecer-se política e ideologicamente nas primeiras décadas da República. Profissionais e estudiosos de áreas do conhecimento bastante heterogêneas, tais como medicina, engenharia, direito, artes, letras e história, buscavam entender e resolver o grande problema do “atraso nacional”, indicando a necessidade de investigar as razões que inviabilizavam a “modernização” do país. Conforme destaca Ângela de Castro Gomes (2010), tais intelectuais apropriaram-se de maneira bastante significativa da “missão” de modernizar a sociedade brasileira, que, na perspectiva desses sujeitos, ainda era fortemente marcada pelo “atraso” deixado pelo regime monárquico e escravista há pouco tempo deposto.

Nesse contexto, é necessário destacar a clara relação de antagonismo entre a oligarquia acciolyna, que comandava a política cearense nessa conjuntura, e a ação social e postura política de Rodolfo Teófilo. Diante da inexistência de políticas de saúde pública efetivas, bem como de assistência social fornecida pelo estado, o farmacêutico destacou-se, por meio de sua iniciativa particular, como um dedicado agente proativo na área da saúde pública. Também foi relevante a constante denúncia dessa ineficiência política, que para ele refletia uma doentia falta de vontade dos detentores do poder local em fazer a terra e sua gente verdadeiramente prosperar, pois se digladiavam e se aliavam com a mesma facilidade quando entravam em jogo as possibilidades de favorecimento ou de prejuízo de seus interesses particulares, deixando o povo à deriva, conforme a maré de suas ambições (Soárez, 2009).

Desse modo, conforme ressalta Maia (2022), Teófilo empreendeu suas atividades sem esconder sua insatisfação com a maneira com que a saúde pública era encaminhada pelas autoridades locais. Apresentava denúncias em jornais locais, sempre a enfatizar que acreditava mais na iniciativa particular do que no poder público, que, sem sua visão, estava tomado por homens que só visavam satisfazer seus interesses e os de seus aliados (Maia, 2022).

É no âmbito dessa conjuntura nacional que emerge a figura de Antônio Pinto Nogueira Accioly, que, empossado no cargo de governador em 1896, deu início a uma política oligárquica duradoura no Ceará, onde, por 16 anos (1896-1912), lançou mão, reiteradamente, de práticas nepóticas e corruptas, além de reconhecida truculência contra seu crescente número de adversários políticos, entre os quais Rodolfo Teófilo, a fim de manter-se no poder (Porto, 1993).

Sabendo desse contexto em que se inserem o autor e sua narrativa que ora analisamos, constante da obra *Varíola e vacinação no Ceará*, entendemos que, nesse documento, muito mais do que a simples descrição de sua empreitada individual pela imunização da população cearense contra a grave enfermidade, Teófilo manifesta a negligência governamental refletida na ausência de um aparato profilático contra enfermidades (Vasconcelos, 2023).

O governo do Estado … não cuidou de mais lazaretos e deixou que a varíola tomasse conta da cidade. Não foi preciso muito tempo para esta peste fazer da bela e risonha Fortaleza uma cidade impossível de se visitar, e mais, de nela se viver. Desde que não havia mais para onde levar os bexigosos, começaram a ficar eles em seus próprios ranchos, que, como já disse, eram nos subúrbios e onde havia árvores no próprio centro da capital. … Indiferentes eram os poderes públicos, indiferentes eram os particulares à sorte destes miseráveis. … Abandonados completamente se viram os variolosos dentro de uma cidade com foros de civilizada (Teófilo, 1910, p.53-54).

Sem o comprometimento e o interesse com a manutenção dos arranjos do poder oligárquico local, sua narrativa e sua ação social, ainda que benéficas ao povo, eram percebidas pelos gestores da situação a partir da perspectiva da disputa por poder. Nessa esteira, pouco importava se as denúncias eram fundamentadas e se o trabalho desempenhado era, ou não, de grande valor para sanar um histórico problema de saúde pública, como a varíola.

Nesse sentido, vale destacar que Teófilo buscou por diversas tentativas orientar os poderes constituídos acerca de como deveriam proceder a fim de efetivamente prevenir o alastramento da doença. Segundo ele, a varíola era um “mal congênito da terra cearense” (Teófilo, 1910, p.5), característica que fazia urgir a necessidade de medidas efetivas e permanentes de prevenção e controle dessa enfermidade, tal como a imunização em massa, estratégia que tornava imprescindível que a vacina fosse produzida no Ceará e em larga escala.

O farmacêutico, decidido a empreender de maneira voluntária a vacinação em massa da população cearense, mas prevendo as dificuldades que enfrentaria em fabricar e disponibilizar a linfa na quantidade necessária para imunizar grande contingente de pessoas, buscou a ajuda do então presidente do estado, o médico Pedro Borges, para a implementação de um instituto vacinogênico no Ceará. Todavia, o político, alegando a hostilidade do clima, julgou descabida a empreitada, embora louvasse sua boa vontade, prometendo-lhe, inclusive, o título de benemérito, caso alcançasse sucesso nessa missão. Deixou claro, no entanto, que ele não esperasse qualquer custeamento por parte dos cofres públicos locais para esse trabalho (Lira Neto, 1999).

A completa recusa em ao menos tentar viabilizar a criação de um instituto vacinogênico no Ceará refletia, aos olhos de Teófilo, mais uma demonstração da pouca importância que, em sua opinião, a atual gestão parecia relegar à saúde pública. Diante do crescimento do número de casos endêmicos de varíola na cidade, encaminhando-se para uma nova epidemia, a estrutura profilática estava ainda mais precária do que no momento do último grande flagelo, em 1878, sobretudo após o fechamento dos já insuficientes equipamentos destinados ao isolamento e cuidado dos enfermos, como o Lazareto da Lagoa Funda:

Pode-se bem avaliar o grau de nossa civilização e o valor que tinha entre nós a saúde pública. … Foi nesse tempo cheio de angústias para a população retirante que o governo do Estado entendeu acabar com o único favor a esta infeliz gente, o tratamento de algumas dezenas de bexigosos no Lazareto de Lagoa Funda, mandando fechá-lo. Esta medida a todos abalou. Fechar um hospital em tempo de epidemia e tal ordem emanar de um médico, do mesmo homem que na mocidade havia dado seu esforço, toda a sua ciência, que foi um abnegado enfim, a variolosos no mesmo lazareto, que agora manda tão friamente fechar! (Teófilo, 1910, p.58-59).

Diante da ausência do fornecimento, por parte do estado, dos mínimos subsídios para a missão idealizada por Rodolfo Teófilo, não foram poucos os desafios para o sucesso da vacinação da população cearense, iniciada em janeiro de 1901. O farmacêutico procedeu à compra de todo o material necessário, incluindo dois bezerros, para a produção da linfa em sua própria residência – depois de ter assistido a algumas sessões de preparo e aplicação da vacina no Instituto Vacinogênico de Salvador e após incansáveis tentativas de inoculação de linfas encomendadas a diferentes vacinogênicos (Salvador, Rio de Janeiro e São Paulo) (Teófilo, 1910).

Concluída a produção da vacina, Teófilo convocou uma plateia de médicos para assistir a todo o processo. Com isso, desejava comprovar seu feito, mostrando a todos sua viabilidade, bem como vacinar voluntários com credibilidade científica para a divulgação de seu trabalho. A estratégia surtiu efeito, pois, com o comparecimento do próprio presidente do estado, o doutor Pedro Borges, para se vacinar, por exemplo, muitos se sentiram encorajados a procurar a vacina (Lira Neto, 1999).

Dessa maneira, após conseguir produzir o imunizante com seus próprios recursos e aparatos e obter o reconhecimento por parte do governo, os primeiros desafios haviam sido superados. Diferentemente do que se poderia supor, no entanto, esses, de longe, não seriam os principais obstáculos a transpor. Era ainda “necessário captar a confiança de muitos, mover os indiferentes a aceitarem o salutar preservativo e convencer os antivacionistas de sua obstinação no erro” (Teófilo, 1910, p.99). Para Teófilo, mais difícil que convencer a “arraia miúda” seria convencer os “ignorantes com rótulos de ilustrados” (p.99). Mais tarde, outro grande obstáculo surgiria, o de lidar com a difamação promovida pelo próprio governo ao trabalho social por ele desenvolvido, conforme observaremos adiante.

Sem contar com qualquer parceria governamental, apenas com a ajuda de alguns poucos profissionais da saúde que se voluntariaram a vacinar os moradores que buscassem a vacina nos locais previamente anunciados pelo farmacêutico nos jornais do estado, Rodolfo Teófilo percorria os subúrbios a cavalo, a fim de vacinar aqueles que jamais receberiam o antídoto de outra forma. Naquele contexto, conseguiu, no fim de 1901, vacinar 3.585 pessoas em Fortaleza, conforme os dados apresentados no jornal *A República* ^4^ (Teófilo, 1910, p.142).

Ao publicizar os números alcançados por seu trabalho nos jornais, Teófilo sempre ressaltava que muito mais poderia ser alcançado se houvesse apoio do governo, sobretudo no sentido de colaborar para a conscientização da população acerca dos benefícios da vacina ou mesmo por meio de medidas mais enérgicas, como, por exemplo, fazer executar a lei n.13/1891, que tornava a vacinação obrigatória. É o que observamos no seguinte trecho da publicação de 1 de outubro de 1901 do jornal *A República*:

Como se vê a varíola está quase extinta dos subúrbios a oeste de Fortaleza e podia estar de todos se os Poderes Públicos secundassem os nossos esforços, pondo em execução as leis que tornam a vacina obrigatória. Os nossos bons desejos levando a vacina aos domicílios não bastam para demover o povo do propósito de não se vacinar. O preconceito contra este poderoso profilático tem profundas raízes, que se não extirpam a não ser pela força ou pela instrução. Com um pouco de paciência e de retórica temos conseguido muito, mas tudo por aquele meio é quase impossível. O governo venha em nosso auxílio que levaremos ao cabo a nossa missão (Teófilo, 1910, p.134).

Em resposta a essa publicação, no dia seguinte, 2 de outubro de 1901, o mesmo jornal, que era o diário oficial do governo, demonstrou a insatisfação com o teor de “cobrança” observado no texto de Teófilo. A resposta informava que, a partir das afirmações do farmacêutico, podia-se erroneamente inferir que os poderes públicos do estado quedavam-se indiferentes “ante um mal que tantas vítimas tem feito”, e alegava que, contrariando as palavras do benemérito, muitos haviam sido os esforços empreendidos pelo governo do Ceará na tentativa de evitar a propagação da varíola e de outros *morbus* que costumam aparecer em determinadas épocas do ano. No entanto, não exemplificava os supostos trabalhos desenvolvidos nesse sentido. A publicação afirmava que fugia das competências do governo o trabalho de “obrigar” a população a aceitar a vacina por ele ofertada, pois não poderia interferir na liberdade dos cidadãos. É o que se demonstra a partir do excerto abaixo:

O que o governo não pode fazer é obrigar a nossa população a se vacinar, quando ela tem, infelizmente, o espírito imbuído de preconceitos arraigados contra esse meio profilático, nem basta o instinto da própria conservação a adverti-la do perigo [a] que assim se expõe. Ninguém de boa-fé poderá negar os bons serviços prestados pelo ilustre Sr. Rodolfo Teófilo à população desta cidade, propagando a vacina sem nenhuma retribuição; mas, por igual, não será justo contestar-se a ação benéfica dos Poderes Públicos, neste como em outros assuntos, sobre que tem naturalmente de se voltar a sua atenção (Teófilo, 1910, p.135).

Até o dia da mencionada publicação, o trabalho desenvolvido pelo farmacêutico, ainda que não tivesse contado com o auxílio dos poderes públicos, gozava de algum reconhecimento por parte do governo e não sofria qualquer tentativa de impedimento. Vale dizer, como destaca o texto de Maia (2022), que o referido doutor Pedro Borges, inicialmente, demonstrou grande simpatia à campanha de Teófilo, como é possível observar neste trecho de sua fala de abertura da primeira sessão da legislatura da Assembleia Provincial em 1901:

Registro com satisfação e louvor o inestimável serviço prestado pelo distinto farmacêutico Rodolfo Marcos Teófilo que, por amor do bem público, se prestou a vacinar gratuitamente a centenares de pessoas, no período agudo da epidemia. Da capital passou a varíola a contaminar diversas localidades do interior, percorrendo Iguatu, Morada Nova, Benjamin Constant, S. Francisco, Missão Velha, 216, Crateús, Ipu, Sobral, Granja, Quixeramobim, Saboeiro, Jardim, Senador Pompeu, Baturité, Vazantes, Quixadá, Coité, Crato e outras. Atendi sem perda de tempo a todas as reclamações sobre ambulâncias e linfa vacínica, concorrendo o governo, pelos meios a seu alcance, para minorar os efeitos dessa epidemia nas localidades onde grassava (Borges, 1901, p.60).

Esse tom elogioso ao trabalho de Teófilo, contudo, foi deixado de lado quando o mencionado presidente do estado selou um acordo político com o então vice-presidente do estado do Ceará durante sua gestão, Nogueira Accioly, pelo qual, em troca do apoio dos deputados aliados a Accioly, dividiria as decisões do estado com seu vice-presidente, o que previa, ainda, a permuta dos cargos entre ambos (Sombra, 1997).

A partir do momento em que o trabalho do farmacêutico começa a tomar corpo, tornando-se mais difícil de ser conduzido sem o auxílio de, ao menos, uma incipiente sistematização ou colaboração financeira por parte do estado, Teófilo vê-se na necessidade de lançar luz sobre a postura omissa do governo na implementação de medidas efetivas de combate à epidemia. Nesse sentido, é notória a mudança de comportamento dos representantes do governo em relação ao trabalho desenvolvido pelo, até então, louvado benemérito.

Fui tolerado enquanto não disse: – preciso de um Cirineu para levar a cruz ao Calvário, e só vós, senhor governo, podeis ajudar-me pondo em execução as leis já criadas, que tornam a vacina obrigatória. … Agora mais trabalhosa ainda seria a minha via sacra. Contudo não esmoreci. Dominado por esta ideia a qual me escravizei, não via que tinha caído no desagrado da gente que governa o Ceará e que não tardaria ela conduzir-me à via dolorosa do insulto (Teófilo, 1910, p.137).

Ou seja, a partir do momento em que Rodolfo Teófilo se coloca contra o que afirmava ser “injustiça”, “irresponsabilidade”, “negligência” do poder público, e toma para si a missão de acabar com a varíola no estado, o governo e seus agentes viram nessa ação um ato panfletário de oposição política e, até mesmo, subversivo.

Além disso, convém ressaltar, conforme aponta Maia (2022), ao analisar a rede de assistência no Ceará, é possível entender que as disputas entre médicos e farmacêuticos puderam ser percebidas com mais intensidade no início do século XX, como reflexo de um período em que a classe médica buscava consolidar-se como detentora oficial das práticas de cura e como principal formuladora de estratégias para o ordenamento da saúde pública.

Quando o farmacêutico publicizava suas suspeitas acerca da linfa utilizada pelo poder público, vinda do governo federal, destacando que parte da população apresentava reações à vacina devido a sua má qualidade e trazendo para si o papel de produzir uma linfa de qualidade no próprio estado, criticava abertamente a postura da Inspetoria de Higiene, chefiada pelo médico Meton de Alencar. Conforme o discurso de Teófilo, Alencar não conseguia alcançar os resultados esperados para a manutenção da higiene pública. Assim, os confrontos entre classes profissionais são trazidos à tona. Isso demonstra que a postura hostil do governo em relação ao trabalho desempenhado por Teófilo, além de refletir posição reativa à crítica contumaz empreendida pelo farmacêutico ao trabalho que julgava insuficiente, negligente e irresponsável por parte do estado, reflete também uma disputa de classes (Maia, 2022).

Os desafios encontrados por Teófilo durante a campanha de vacinação acirraram-se, sobremaneira, a partir de 1903, quando o doutor Meton de Alencar assumiu a Inspetoria de Higiene do Ceará. Formado pela Faculdade de Medicina do Rio de Janeiro, Alencar se manteve à frente da inspetoria até 1912. Como bem aponta Maia (2022), na condição de inspetor de higiene, o médico traduzia o que havia de mais inflamado acerca dos ofícios de cura. Declarava de maneira enfática em seus relatórios oficiais sua grande preocupação com a atuação de outros profissionais da saúde que atuavam no estado. Tratava-se, para ele, de indivíduos sem formação, indicados por “filhotismo ou compadresco”, aparentemente sem critério científico que embasasse suas funções, conforme se observa neste excerto:

Seguindo a errônea praxe antiga, tem surgido ultimamente não só no interior do estado, mas ainda aqui, na capital, indivíduos que consciente ou inconscientemente vão ao abrigo da justiça, exercendo criminosa e ilegalmente, as profissões de médicos, farmacêuticos e dentistas (Alencar, 1904, p.5-6).

Entre suas muitas críticas, o médico questionava o tempo de estudo e a forma como esses profissionais adquiriam seus diplomas e licenças. Nos relatórios, ele enfatizava a necessidade de uma rigorosa fiscalização do serviço prestado, assim como uma regulamentação mais dura para conter o avanço dos atendimentos tidos como ilegais e arbitrários dentro do exercício das artes de curar. Contudo, apesar de afirmar que comprovaria diversos fatos que atestavam a veracidade de suas denúncias, os únicos registros encontrados de acusações feitas pelo doutor Meton de Alencar foram direcionados apenas a Rodolfo Teófilo (Maia, 2022).

Assim, a despeito dos grandes prejuízos ao trabalho de prevenção à varíola no Ceará devido ao posicionamento negligente do governo, nada iria comparar-se à atitude caluniosa e difamatória que a gestão governamental passaria a ter a partir de 1903. Um novo pasquim que circulava por Fortaleza, *O Tempo*, redigido por membros do partido governista, configurado como escancarado desdobramento do jornal oficial *A República*, começou a veicular toda sorte de notícias falsas, sem qualquer compromisso com a comprovação das informações repassadas, sobre a qualidade da vacina produzida/distribuída por Rodolfo Teófilo. Atitude que poderia colocar em xeque todo o trabalho desempenhado até aquele momento.

Vejamos o teor da primeira nota publicada, nesse sentido: “A linfa do Sr. Rodolfo Teófilo é mesmo uma maravilha. De uma criança, sabemos nós, que tendo sido vacinada pela manhã, à tarde era com os anjos. Não resistiu a inocente criaturinha, ao frouxo, que a linfa lhe produziu” (Teófilo, 1910, p.213).

Essa história, completamente inverídica, rapidamente se espalhou por toda a cidade. Por todos os lados, ouviam-se os boatos de que a vacina do senhor Rodolfo Teófilo estava matando as pessoas, sobretudo as crianças. Conforme as memórias do farmacêutico, de tanto que se repetiu a mentira, a história ganhou *status* de verdade entre os populares. Ainda que, após três anos de trabalho com a vacinação da população cearense, não tenha sido registrado um único incidente em decorrência do imunizante produzido e distribuído por Teófilo, a sombra dessa suposta ligação da morte de uma criança com a vacina, aliada ao reforço diário que a publicação de matérias apócrifas incutia sobre o perigo da vacinação empreendida, trouxera muitos danos à campanha de imunização (Teófilo, 1910).

Pouco tempo após o início das publicações do pasquim governista, começa a ser difundida a ideia de que todos os vacinados corriam risco de vida. Os pedidos vindos do interior por novos carregamentos de vacina começaram a escassear, e a vacinação domiciliar passou a encontrar nova e mais forte resistência, visto que, agora, se apoiava em “argumentos reais”, e não mais em temores injustificados.

A cada golpe sofrido por meio das calúnias difundidas, Teófilo rebatia com a apresentação das estatísticas, que tornavam clara a relação da ausência de novos casos na cidade com o número de vacinados. Realidade que se contrapunha a de estados vizinhos, como o Rio Grande do Norte e a Paraíba, que, sem um projeto, ainda que individual, de vacinação em massa, vivenciavam os terrores decorrentes dos surtos da doença (Teófilo, 1910, p.321). Como parte de suas tentativas de defesa, em abril de 1904, o farmacêutico recorreu à publicação de um abaixo-assinado, em que 194 homens, entre os quais figuras ilustres do cenário intelectual e político do Ceará, tais como João Salgado (gerente do Banco do Ceará), Eduardo Studart (juiz) e Eduardo Salgado (médico que foi inspetor de higiene no Ceará), além de sete professores de direito, seis farmacêuticos e oito médicos, o defendiam das acusações contra sua vacina, buscando validar os bons resultados de seu trabalho (Maia, 2022).

No dia 11 de março de 1905, em clara represália ao último relatório mensal publicado pelo farmacêutico em jornal oficial do estado, em que denunciava a irresponsabilidade das autoridades sanitárias em relação ao caso de mais dois doentes desembarcados por vapores de passagem em Fortaleza, o editorial do jornal *A República* publicou uma notícia que relacionava a morte de outra criança à vacina ofertada pelo farmacêutico, dessa vez por meningite.

O referido periódico, embora oficial, não se preocupava mais em manter qualquer verniz de imparcialidade e referia-se ao farmacêutico como ignorante, pretensioso e espertalhão, que insistia em “ludibriar a boa-fé dos incautos com o engodo de sua linfa vacínica” (citado em Teófilo, 1910, p.302). A notícia terminava lançando a ameaça de que rigoroso inquérito seria instaurado para verificar sua responsabilidade na morte da vítima.

Diferentemente do que se podia esperar diante da ameaça de processo de investigação sobre a relação da vacina com a morte da criança por meningite, Teófilo, em vez de acuar-se, passou a exigir que tal apuração realmente acontecesse. Sabia que nada seria feito, pois não era intenção do governo proceder a qualquer esclarecimento dos fatos, visto que a única motivação para a propagação dessas notícias consistia na vontade de difamar um importante trabalho social, do qual, criminosamente, o governo não participava e não tinha nenhum argumento plausível para justificar essa indiferença.

Reptei o governo para que mandasse sindicar do fato e apurasse a minha responsabilidade. Até hoje o poder público não deu um passo no terreno da lei e da justiça. … Se é exato o que diz o órgão oficial a respeito do perigo que corre a saúde pública de Fortaleza, onde a população é diariamente intoxicada pelo vírus vacínico por mim preparado, que faz o governo que não impede esse atentado contra a vida dos habitantes desta capital? Não precisa mais do que uma ordem do Sr. inspetor de higiene para que me submeta (Teófilo, 1910, p.306-307).

Como previsto por Teófilo, o famigerado inquérito não ocorreu, mas sim novas enxurradas de acusações, jamais acompanhadas de comprovação. Estampando com cada vez mais frequência as páginas do jornal oficial, bem como dos pasquins governistas, as notícias difamatórias acerca da vacina e da pessoa de Rodolfo Teófilo, que, de “benemérito”, “homem de notório saber”, passava, então, a ser chamado de “irrisório filósofo”, “higienista-mirim” e “charlatão”, acabaram por desestabilizar seriamente o trabalho do farmacêutico, já de difícil execução (Teófilo, 1910).

A gente do governo não sustenta uma discussão no terreno científico. Atira-se-lhe a luva; não a apanha, recua, mas insultando. Não é que entre ela não haja homens cultos, muito poucos é verdade, ... é que discutir assuntos científicos não é do seu programa, uma vez que seja respeitada a individualidade do adversário. Quer a ofensa pessoal que se retalhe o inimigo até no lar, e quando não houver mais pele para ser arrancada pelo látego da calúnia, exponham-se-lhe as moléstias que sofre, como se as enfermidades fossem um delito! (Teófilo, 1910, p.440).

Sem se conformar com a ideia de que as suspeitas infundadas a respeito da linfa por ele produzida permanecesse sem resposta, vilipendiando a memória de seu trabalho, a suas próprias expensas, enviou dez tubos da vacina ao Instituto de Manguinhos, no Rio de Janeiro. Em 10 de maio de 1907, o laudo oficial do exame, assinado pelo doutor Figueiredo Vasconcelos, atestou não ter sido encontrada na linfa a presença de nenhum micro-organismo prejudicial à saúde, e acrescentava que, como forma de verificar a eficácia da vacina, o instituto utilizou as doses em crianças, as quais apresentaram os melhores resultados possíveis (Teófilo, 1910, p.354-356).

Dessa maneira, por meio da aprovação de uma instituição “acima de qualquer suspeita”, Rodolfo Teófilo comprovou a qualidade de seu imunizante e fez a todos saber no *Jornal do Ceará*, anunciando que continuaria a vacinar gratuitamente em sua residência, todos os dias, entre uma e quatro horas da tarde, “enquanto o Sr. inspetor de higiene não determinasse o contrário” (Lira Neto, 1999). Destarte, sem subsídios que permitissem a continuação de sua estratégia negacionista acerca da qualidade da vacina ofertada pelo farmacêutico, o governo Accioly não encontrou justificativas para mandar fechar o vacinogênico da rua Visconde de Cauípe, e passou a calar-se quanto ao trabalho que Teófilo continuou a desempenhar até o fim da vida, pois, conforme tantas vezes alardeou, a vacinação não poderia parar.

## Da varíola à covid-19: guerras de narrativas em razão da obrigatoriedade da vacina

Em 1905, matéria publicada no *Diário Pernambucano* narrava o percurso pedregoso trilhado por Rodolfo Teófilo para empreender a vacinação contra a varíola no Ceará, destacando os bons resultados alcançados por sua cruzada, que conseguira erradicar a doença no estado, após cerca de quatro anos, e indagava: “E depois do que praticou e escreveu Rodolfo Teófilo, ainda haverá no Brasil quem faça guerra à vacina obrigatória?” (citado em Teófilo, 1910, p.275). O jornalista que elaborou tal matéria certamente não poderia supor que, passados mais de cem anos, seu questionamento permaneceria atual no discurso de sujeitos vivenciando os horrores de uma nova doença infectocontagiosa, mortal e de alto impacto social, a covid-19. Assim como ele, não conseguiriam compreender a oposição governamental à principal solução para debelar a crise sanitária, a vacinação.

Nos meses iniciais de 2020, eram incertas as formas de transmissão da covid-19, suas formas de atuação nos diferentes organismos acometidos e a terapêutica adequada. Também não havia imunizantes. Por meio do relato de um médico que atuava na linha de frente contra a covid-19 na cidade de Fortaleza, epicentro da doença no Nordeste naquele ano, o estudo de Lima e Lima (2022), que compõe um capítulo do décimo volume da coleção *Uma história brasileira das doenças*, buscou refletir acerca dos impactos causados pela doença num dos períodos mais críticos de seu enfrentamento. Vejamos um trecho do depoimento de Francisco Edilson Aragão Junior, médico, analisado pelo referido estudo:

Em um cenário de incertezas, seguimos, o mundo segue em busca de uma vacina e de um tratamento eficaz contra a pandemia causada pelo novo coronavírus, na expectativa de simplesmente responder à seguinte pergunta: ‘Até quando?’ … Fica a esperança de dias melhores (Lima, Lima, 2022, p.287).

A partir do que se pode avaliar na narrativa do sujeito que protagoniza o referido estudo, era notória a ideia de que a descoberta da vacina seria a grande alternativa para solucionar o problema da pandemia, a grande alternativa que nos possibilitaria esperar por dias melhores. A hipótese mostrou-se acertada meses depois, pois apenas por meio da implementação da vacinação em massa a doença começou a ser efetivamente controlada. Contudo, o referido médico não contava que o desafio não consistiria apenas em descobrir um imunizante para aquela enfermidade que, no Brasil, chegou a levar a óbito quase duas mil pessoas em um único dia (Valente, 3 mar. 2021). Haveria, além do obstáculo científico, outro que se mostraria ainda mais perturbador, o uso político-ideológico de uma causa sanitária, em que a vacina passaria a ser irresponsavelmente descredibilizada por segmentos de apoio ao governo federal, em especial, à pessoa do presidente da República, Jair Messias Bolsonaro, trazendo prejuízos incalculáveis à gestão da pandemia e à vida dos cidadãos.

As testemunhas oculares de ambos os flagelos, ainda que separadas por mais de cem anos, ficariam igualmente estarrecidas ao perceber as desconcertantes similitudes envolvidas nas motivações que permearam o conjunto de práticas políticas em tentativa de dificultar a implementação da vacinação em massa nas duas ocasiões. Após explanar brevemente o contexto de oposição governamental à vacinação contra a varíola no Ceará, apresentaremos um panorama semelhante acerca da pandemia de covid-19 no Brasil.

O início do segundo ano de governo do então presidente Jair Bolsonaro (2019-2022) foi marcado pela chegada do novo coronavírus (SARS-CoV-2) no território nacional. Desde o início, o governante assumiu postura que refletiu sua pouca importância conferida à doença e aos possíveis riscos à vida dela capazes de decorrer. Diante da confirmação da circulação do vírus no país, afirmou, por conta própria, que a situação não era “alarmante” (Albuquerque, 7 jul. 2020), pois a doença não seria mais do que uma “gripezinha”, e todo o alarde engendrado pela mídia não passava de “fantasia”. Assim, nos seis meses posteriores à confirmação do primeiro caso no Brasil, mesmo diante do fato de o país ter-se tornado o epicentro da pandemia, e com mais de 117 mil mortes nesse período (Gonçalves, 27 ago. 2020), Bolsonaro continuou a desdenhar da patologia e a rejeitar a gravidade da situação.

A condução negacionista e desajustada do presidente no combate à pandemia, que impossibilitava um direcionamento nacional unívoco de combate à doença, não se restringiu a sua retórica presidencial, mas evidenciou-se, sobremaneira, na recusa às orientações científicas de prevenção e enfrentamento da enfermidade. Foram recorrentes as aparições públicas do governante sem máscara, estimulando aglomeração entre seus apoiadores, além de incitar, nesses eventos, a insubordinação popular às ações organizadas por parte dos governadores para o enfrentamento da covid-19, que, alinhadas às recomendações científicas mundiais, preconizavam como principal ferramenta de controle o isolamento social horizontal^5^ – paralisação parcial do transporte público e fechamento do comércio, com exceção dos serviços considerados essenciais. Porém, segundo o discurso do presidente, a “economia não podia parar”, e, diante de uma grave crise econômica, ele não teria o que fazer. Dessa forma, de acordo com sua narrativa, “infelizmente algumas mortes seriam inevitáveis, mas era preciso tocar o barco” (Infelizmente..., 27 mar. 2020).

Vale destacar que o discurso proferido por Bolsonaro reverberava o próprio discurso de seus apoiadores, que, de suas mídias digitais, defendiam e financiavam campanhas contra a quarentena e a favor do isolamento vertical, uma alternativa ilusória de enfrentamento à pandemia sem nenhum respaldo científico, que visava, principalmente, suavizar uma total indiferença às inúmeras mortes, pois, para esses grupos, muito pior seria um colapso econômico (Frigotto, 2020). Tanto que, entre os dias 27 e 29 de março de 2020, empresários de todo o país, especialmente dos setores de comércio e transportes, financiaram a realização de carreatas em quase todos os estados do Brasil, em apoio à campanha “O Brasil não pode parar”. No entanto, tendo sido proibidas pela Justiça as principais formas de manifestação desses apoiadores, as chamadas “carreatas da morte”, poucos foram os que se identificaram (Bortone, Hoeveler, 7 abr. 2020).

Sem se importar com a função de um presidente num país democrático, Bolsonaro jamais buscou dialogar com as diferenças. Governando apenas para “os seus”, sempre utilizou a estratégia de deslegitimar os anseios e as demandas sociais daqueles que, pelas mais variadas razões, não eram coniventes com a ideologia circular e fechada de extrema direita que ele representa(va). Bolsonaro desconsiderava as contradições, os conflitos, a diversidade de necessidades e de interesses do “povo”, que, conforme Laclau (2013), tem a heterogeneidade como sua principal condição.

Tal posicionamento não foi diferente na gestão da pandemia. Sem habilidade para conduzir tamanha crise sanitária e com total insensibilidade e falta de solidariedade com o sofrimento de milhares de doentes que agonizavam sem oxigênio nos corredores dos hospitais, com o número alarmante de mortes diárias, em que braços e máquinas eram insuficientes para a abertura de tantas covas, bem como com o esgotamento físico e emocional dos profissionais da saúde que chegavam a morar nos hospitais, a fim de tentar suprir a imensa demanda e como estratégia de proteção a seus familiares (Sant’anna, 2021), o então presidente do Brasil estrelou cenas e decisões deploráveis na condução política do fenômeno patológico.

Assim, após as duas “tentativas” de colocar profissionais, em tese, qualificados para assumir o Ministério da Saúde (os médicos Luiz Henrique Mandetta e Nelson Teich, demitidos por discordar dos direcionamentos do presidente), a pasta passou a ser administrada por militares, Eduardo Pazuello e, posteriormente, Marcelo Queiroga. Apoiaram incondicionalmente as deliberações do presidente durante todo o restante de seu mandato, sobretudo na promoção do uso da cloroquina e da hidroxicloroquina como opções de “tratamento precoce”, fármacos utilizados há muitos anos no tratamento de enfermidades como malária, lúpus e artrite reumatoide, mas que não obtiveram qualquer comprovação científica como alternativa de tratamento para covid-19 (Ektorp, 2020).

Sem poder contar com o embasamento científico que justificasse a defesa dessa terapêutica, Bolsonaro não hesitou em recorrer a supostas “informações alternativas”, embasadas nas opiniões de um restrito corpo médico aliado, para afirmar que “a cloroquina estava dando certo de forma não comprovada cientificamente” (Albuquerque, 7 jul. 2020). Afirmava ele que seus opositores – governadores e prefeitos que se guiavam pelas orientações da ciência – estavam utilizando a pandemia como arma política para desestabilizar a economia de seu governo, por meio do recrudescimento do isolamento social e pela recusa em implementar o uso dessas medicações, sem se importar com a vida dos brasileiros que desejavam “tentar a cura” por meio do uso desses remédios (Albuquerque, 7 jul. 2020).

Dessa maneira, como destaca Cesarino (2021b), em vez de investir-se da missão de promover condições para que seus governados lutassem pela vida ancorados em intervenções amplas e planejadas, a conduta do presidente Bolsonaro naturalizava a ideia de que a luta contra o vírus consistia em uma guerrilha caótica, sem comando central, em que os cidadãos deviam desenvolver suas próprias estratégias de sobrevivência, contando, em última instância, com a vontade inescrutável de Deus.

Assim como Donald Trump, então presidente dos EUA, cujo comportamento político procurou imitar, Bolsonaro afirmou reiteradas vezes que os direcionamentos da própria OMS, relacionados ao uso desses medicamentos para covid-19, davam-se por “motivações ideológicas”. Dessa forma, incutiu um valor político na “escolha” dos indivíduos por utilizarem ou não tais fármacos, fazendo, inclusive, diversas anedotas acerca do assunto, tais como “quem é de direita, toma cloroquina, quem é de esquerda, tubaína!” (Bertolli Filho, 2021).

Esses posicionamentos podem ser interpretados dentro do âmbito teórico da pós-verdade, no qual, segundo Harsin (20 dez. 2018), é fundamental a divulgação deliberada de dados inverídicos e/ou imprecisos, com o objetivo de moldar as informações para se adequar a uma agenda específica. Haja vista que o então presidente do Brasil, como partícipe desse núcleo de sujeitos que buscavam incutir uma ideia conspiratória nos resultados divergentes de suas expectativas em torno do uso da cloroquina e da hidroxicloroquina, guiou sua gestão no intuito de contribuir com o enriquecimento exacerbado de empresas farmacêuticas. Podemos aferir a intencionalidade contida em suas afirmações sobre essas medicações, que, segundo os dados do Sindicato da Indústria de Produtos Farmacêuticos, tiveram o consumo acrescido em 358% no Brasil durante a pandemia (Veja quem…, 11 jul. 2020).

Além disso, conseguindo apoiar-se na quimera da eficácia dessas medicações como alternativa de tratamento precoce para a patologia, Bolsonaro vislumbrava acabar com a justificativa para o isolamento social horizontal. O isolamento impactava fortemente a atividade econômica e trazia prejuízos a sua popularidade entre seus apoiadores, que, em grande medida, o escolheram como uma aposta neoliberal em matéria econômica, devendo defender a manutenção do mercado, mesmo que em detrimento de muitas vidas.

A despeito desse artifício, como bem relembra Amado (2022), ao se descortinar cotidianamente nos noticiários a agonia dos doentes nos hospitais, cada dia mais cheios, e o número vertiginosamente crescente de mortos, inclusive por falta de atendimento hospitalar, em razão da indisponibilidade de leitos, entre outras causas, a tentativa do presidente de minimizar a importância da enfermidade, debochando, por vezes, daqueles que se preocupavam com ela, não obtinha os efeitos desejados, pois os fatos desvelavam a verdade por si só. Diante disso, cidades de diferentes partes do país manifestavam-se em panelaços contra Bolsonaro durante seus pronunciamentos. Episódios que sinalizavam queda acentuada de sua popularidade (Amado, 2022).

É nesse contexto de insatisfação com as condutas do presidente na gestão da pandemia, centradas na negação, na atenuação da devastação do fenômeno e no descaso em relação aos dramas protagonizados por vítimas e seus familiares que emergem os primeiros lampejos de esperança em relação à produção e à implementação da vacina contra a covid-19 no Brasil. Mais uma vez, a conduta do presidente foi na contramão do que preconizavam os direcionamentos da ciência, colocando-se contra a criação de políticas que viabilizassem a vacinação compulsória, bem como prejudicando as campanhas de imunização, sendo um dos únicos presidentes a afirmar que não se vacinaria, fatos que, conforme salientou o epidemiologista Pedro Hallal em depoimento à CPI da pandemia, foram responsáveis por, pelo menos, 95 mil mortes que poderiam ter sido evitadas (Agência Senado, 24 jun. 2021).

Nesse sentido, é importante recordar que, desde os primeiros meses da pandemia, umas das principais esperanças de os países pobres ou emergentes conseguirem vacinas era por meio da Covax Facility, uma aliança global conduzida pela OMS, o Fundo das Nações Unidas para a Infância, a Aliança Global para Vacinas e Imunização e a Coalizão para Inovações em Preparação para Epidemias, que tinha a finalidade de otimizar o desenvolvimento e a fabricação de imunizantes contra a covid-19 para garantir o acesso igualitário à imunização em todo o mundo (Amado, 2022).

Dezenas de países negociavam para integrar o consórcio, que previa o compartilhamento de informações sobre a doença e o investimento na criação antecipada de uma rede que facilitaria a distribuição em escala das vacinas. Como o consórcio apostava em diversas pesquisas, os países participantes investiriam de uma só vez em várias frentes. Diante dessa convidativa alternativa de busca pelo imunizante, 150 países aderiram à Covax Facility, porém, no Brasil, integrantes da cúpula do Ministério da Saúde manifestavam um estranho desinteresse, com a justificativa de que a adesão ao consórcio seria um arriscado investimento, pois não se tinha a certeza de que a vacina iria realmente ser desenvolvida. Diante do argumento apresentado, a Covax apresentou ao Brasil uma segunda modalidade de adesão, que exigiria um aporte financeiro bem menor, mas, em contrapartida, o país receberia uma quantidade mínima de doses, somente para 10% da população. Em 19 de setembro de 2020, meses depois do início das negociações, próximo de perder o prazo para a assinatura do contrato, Bolsonaro deu o aval para a participação do Brasil na modalidade mais barata.

Além da morosidade em participar do referido consórcio, a cúpula do governo federal, em especial o Ministério da Saúde, condicionado ao autoritarismo de Bolsonaro, que já dera mostras de que descartava facilmente aqueles que não rezassem por sua cartilha, não respondia às muitas outras tentativas de contato para a realização de investimentos para a aquisição de imunizantes, a exemplo do que ocorreu com o Instituto Butantan, que, historicamente, tem sido um dos maiores fornecedores de vacina para o Brasil.

O instituto, desde o início da pandemia de covid-19, associou-se à fabricante chinesa de medicamentos Sinovac Biotech para conceber, desenvolver e testar, em parceria, uma vacina que pudesse impedir o colapso do sistema de saúde brasileiro – a CoronaVac (Cueto, 2020). Cabe destacar que a Sinovac já tinha uma vacina contra o vírus SARS-CoV-1, agente responsável pela epidemia de síndrome respiratória aguda grave na China entre 2002 e 2004, e essa experiência permitiu a adaptação contra o SARS-CoV-2 muito rapidamente.

Em abril de 2020, o Instituto Butantan já testava uma vacina em ensaios pré-clínicos. Esses ensaios em animais mostravam que a vacina tinha grande eficácia em os proteger. Na sequência, começaram os estudos das fases um e dois, com 744 voluntários chineses, que comprovaram sua segurança e eficiência na produção de anticorpos. Esses dados serviram de subsídio para o instituto solicitar o início dos estudos da fase três no Brasil.

A parceria entre as duas instituições (Sinovac Biotech e Butantan) previa troca de conhecimento e de tecnologia, mas a produção da CoronaVac seria local, ou seja, feita totalmente no Brasil. O desenvolvimento da CoronaVac pertencia ao Butantan, utilizando matéria-prima chinesa. Além disso, os estudos clínicos de aplicação da CoronaVac no Brasil também seriam de responsabilidade do Butantan. Por meio dessa parceria, o instituto, antecipadamente, em comparação a outras instituições de pesquisa científica, contava com um estoque prévio de seis milhões de doses de vacina e outras 4,8 milhões em processamento no início do segundo semestre de 2020 (A parceria…, 18 jan. 2021).

Assim, o Brasil poderia ter sido o primeiro país do mundo a fornecer a vacina contra covid-19, visto que o Butantan havia se prontificado a produzir sessenta milhões de doses para o último trimestre de 2020 e mais cem milhões de doses em 2021, mas ficou sem resposta do governo, que ignorou suas seis tentativas de fornecimento de CoronaVac. Um silêncio perturbador diante do clamor por esperança e da agonia de tantos brasileiros (Agência Senado, 27 maio 2021).

Apenas em dezembro de 2020, cerca de quatro meses após os primeiros contatos do Instituto Butantan com o Ministério da Saúde, equipes técnicas da Agência Nacional de Vigilância Sanitária (Anvisa) visitaram o complexo fabril da Sinovac, na China. O objetivo era que o órgão regulador brasileiro averiguasse a produção dos insumos para a vacina. Após a visita, a Anvisa concedeu à Sinovac a Certificação de Boas Práticas de Fabricação de Medicamentos, o que permitiu o início da produção das vacinas no Butantan.

Diante do claro desinteresse do governo federal em adquirir a CoronaVac, optando por postergar o início da vacinação para março de 2021, data de entrega de doses de outros fabricantes, João Dória, então governador do estado de São Paulo, anunciou que a vacinação naquele estado seria iniciada em janeiro de 2021, com a vacina do Butantan.

Quero reafirmar que em São Paulo, de forma responsável, seguindo e observando rigorosamente a lei, no próximo mês de janeiro, cumprindo o protocolo com a Anvisa e obedecendo aos princípios de proteção à vida, vamos iniciar a imunização dos brasileiros. … Se o Ministério da Saúde tiver juízo, competência e a visão de que a vacina deve ser para todos os brasileiros, poderá oferecer também a outros estados a vacina CoronaVac, a vacina do Instituto Butantan, para imunizar brasileiros de outros estados do país (Veja quais…, 24 dez. 2020).

Após inúmeras acusações infundadas por parte de Bolsonaro para tentar desqualificar a aposta daquele que, no momento, adquiria o contorno de principal opositor político em futuro pleito presidencial, no dia 17 de janeiro de 2021, em São Paulo, foi dado início à vacinação contra a covid-19.

De tudo o que observamos neste tópico, o que se apresentou de maneira evidente foi que a vacinação contra a covid-19 foi permeada por controvérsias que a precederam no contexto histórico-político brasileiro. Desde o início da pandemia, Bolsonaro travou embates quase diários contra governadores e prefeitos pela condução de medidas para conter o vírus. Por razões outras que não a preocupação com a vida dos brasileiros, o presidente utilizou vis estratégias para legitimar suas escolhas e desqualificar os direcionamentos de enfrentamento que não coadunavam com os seus. Dessa maneira, podemos depreender que a pandemia deixou de ser uma causa apenas sanitária, configurando-se em conflito político, no qual o principal gestor da nação permitiu que seus próprios interesses se sobressaíssem ao interesse pela manutenção da vida dos brasileiros. O serviço à estrutura desumanizante que o presidente da República sempre desempenhou deixou isso claro.

## Considerações finais

Apesar das abismais diferenças entre os dois contextos analisados no estudo, observamos que, em ambas as situações, as estratégias de legitimação de ações de combate às crises sanitárias estiveram intrinsecamente relacionadas à proliferação de notícias falsas, visando deslegitimar qualquer trabalho que, mesmo indiretamente, denunciasse a irresponsabilidade das respectivas gestões com a vida de seus governados diante da emergência de uma grave doença.

Ambas as gestões das crises sanitárias em tela utilizaram recursos similares para inserir conteúdos negativos na imagem daqueles que deveriam ter a confiança dos cidadãos, simplesmente por ser opositores políticos, e não por estar certos ou errados acerca dos posicionamentos escolhidos para lidar com a emergência de enfrentar os fenômenos patológicos postos.

Nessa batalha, a produção e a veiculação de materiais fraudulentos, com o objetivo de defender determinadas condutas, ainda que sabidamente erradas, bem como de vilipendiar as certas, foram escolhidas como as principais armas. Dessa maneira, os dois governantes, no exercício de suas funções, apropriaram-se da política do escândalo, a qual, como bem descreve e teoriza o sociólogo John Thompson, citado em Castells (2018), projeta uma cortina opaca, a fim de fazer desaparecer ou desviar, ao máximo, a atenção para os debates de fundo e que, verdadeiramente, os comprometem.

Quando esse tipo de estratégia é utilizado na condução de eventos mórbidos de grandes proporções, em que as formas de enfrentamento vão de encontro aos interesses dos governantes, a manipulação dos dados e das informações repassadas à população relativas às formas possíveis de combater as doenças toma contornos ainda mais dramáticos, pois joga com a vida e com a morte de imenso contingente de indivíduos que, crendo ser os governantes responsáveis pela gestão da saúde pública os primeiros interessados em usar todos os mecanismos possíveis para o controle dessas crises, tendem a guiar-se pelas informações por eles repassadas e defendidas.

No primeiro contexto, o representante do estado fabricou inverdades favoráveis ao exercício de seu governo, visando à manutenção irrestrita de seu poderio, tendo utilizado especificamente dois jornais (*A República* e *O Tempo*) aliados de seu governo. No segundo, a proliferação de *fake news* se valeu de uma elaborada rede de produção, que contou com todos os recursos tecnológicos disponíveis para fazer difundir as informações desejadas, não apenas sobre a questão da vacinação e do verdadeiro impacto da pandemia de covid-19 no Brasil, mas sobre os mais variados temas que, de algum modo, beneficiariam o exercício do projeto de governo do então presidente Bolsonaro (Amado, 2022). Assim, observamos que, nas duas situações, em vez de os governantes utilizarem a estrutura da máquina pública para fazer circular informações que contribuíssem para o enfrentamento efetivo das crises que deveriam gerir, utilizaram-na para confundir a população, descredibilizando, nas duas ocasiões, a principal ferramenta que se dispunha para a contenção de ambas as doenças, a vacinação.

Há muito se tem estabelecida a importância da aquisição e divulgação da informação como estratégia de enfrentamento das enfermidades; no entanto, quando voltamos nossa atenção para o modo como esses dois graves fenômenos patológicos foram geridos, deparamo-nos com atitudes, ações e comportamentos que se contrapõem drasticamente a essa compreensão. Tudo se refere ao negacionismo e ao que se relaciona a ele, tanto no que diz respeito a negar a existência de uma patologia epidêmica (Rosemberg, 1992) quanto a negar orientações científicas, divulgando notícia ou informação que as contraria fervorosamente, ainda que sem qualquer forma de comprovação.

Dessa maneira, observamos que, em diferentes momentos da história, a proliferação de notícias falsas foi recorrente, em virtude de sua capacidade de moldar o que se toma por realidade e, assim, beneficiar projetos de manutenção de poder obstaculizados por ameaças, opositores e desafetos políticos. A disseminação de notícias falsas com o objetivo de obter vantagens ou prejudicar alguém de maneira mais ou menos imediata, que atualmente se convencionou chamar de *fake news*, pode parecer recente, mas o sentido, o significado e a intencionalidade por trás da prática já existiam muito antes da invenção da internet e da rede mundial de computadores. Mentiras e manipulações da verdade com alta disseminação social são velhas conhecidas do jogo político mundial.

Outra semelhança que vale ser apontada é que essa postura de enfrentamento dos fenômenos patológicos foi executada em governos com políticas extremamente negligentes em relação à situação dos mais vulneráveis socialmente, fato que se torna nítido com a severidade dos danos entre as populações mais pobres em ambas as ocasiões aqui analisadas. No caso da epidemia de varíola, foram os retirantes “fugidos da seca”, que se avolumavam em abarracamentos insalubres, e moradores dos subúrbios da Fortaleza do início do século XX (Teófilo, 1910); na pandemia de covid-19, no Brasil, foram as populações indígenas e negras, os moradores de favelas e de periferias, os portadores de doenças crônicas e os mais velhos (Gragnani, 12 jul. 2020). Aspectos que apontam para uma continuidade de um recorte de classe na determinação da contaminação e da mortalidade durante as graves crises sanitárias, reforçando a dinâmica das desigualdades no Brasil.

Em ambas as situações analisadas, os dois governantes, movidos pela vontade de fazer valer a própria inércia, ou mesmo incompetência, e por que não dizer desumanidade, diante de crises tão desafiadoras como as sanitárias que se deflagraram em seus governos, não hesitaram em retirar da população sua principal ferramenta no combate às doenças: a informação. Fosse por meio de ocultação de dados e estatísticas, fosse por deturpação da verdade, ou mesmo pela invenção das mais escabrosas mentiras a respeito das possíveis estratégias efetivas de contenção das enfermidades, ambos os gestores, ao avaliar seus governos como simples extensão das próprias aspirações e desejos, não levaram em conta em suas balanças egocêntricas o peso dos danos que tal estratégia teria.

## NOTAS

1 Rodolfo Marcos Teófilo (1853-1932) foi um intelectual multifacetado de grande importância sociopolítica e cultural no Ceará, sobretudo nas duas últimas décadas do século XIX e três primeiras do XX. Neste texto, debruçamo-nos sobre sua atuação como farmacêutico, pois, em vista de seus conhecimentos farmacológicos e de sua qualidade de estudioso inveterado, foi capaz de produzir às próprias expensas a linfa vacínica contra a varíola. Contudo, vale destacar que Rodolfo Teófilo teve ativa atuação em diversos segmentos, inclusive no literário, no qual deixou vasta obra pertencente a diferentes gêneros textuais: romance, conto, poesia, bem como material documental, jornalístico e memorialístico. Tomou parte nos movimentos literários do Ceará, tendo pertencido, desde 1894, à Padaria Espiritual, entidade de fins literários e artísticos que se fundara em Fortaleza, em 1892, uma espécie de agremiação literária que, pelo comportamento irreverente de seus membros, antecipou o modernismo no Brasil. Participou, ainda, de outras agremiações, como o Instituto Histórico e Geográfico do Ceará, o Clube Literário e o Centro Literário. Além disso, participou ativamente da campanha abolicionista no Ceará, que foi a primeira província brasileira a declarar a liberdade dos cativos. Teófilo, ao longo de sua vida, testemunhou diversos episódios de grandes secas (1862, 1877, 1890, 1900, 1915 e 1919), nas quais proliferavam diferentes pestes, tais como tifo, febre amarela, cólera e varíola. Tal experiência o atravessou de maneira bastante significativa, tanto que muito de sua obra literária tem o fenômeno da seca e os seus desdobramentos sociais, como a eclosão de graves epidemias, por exemplo, como parte de suas principais temáticas, aspecto que contribui para que fosse inserida na estética literária regional-naturalista. O escritor, professor e farmacêutico nascido em Salvador-BA, foi para o Ceará, onde viveu a maior parte de sua história, com pouco mais de um mês de vida. Por essa razão, e pelo grande amor que dizia nutrir pela terra que o adotara, definia-se como baiano por acidente e cearense por convicção. Boa parte de sua obra literária, a qual sofreu duras críticas de seus pares, tinha muito mais compromisso em denunciar ao sul do país as condições degradantes a que eram submetidos milhares de cearenses – que, periodicamente, durante as secas, viam-se na contingência de abandonar seus lares em busca de mínimas condições de sobrevivência na capital – do que com a estética e adequação a escolas literárias. Além da escrita literária, Teófilo assinou vasta produção de caráter histórico e sociológico, que se apresenta, por exemplo, em seus livros *A sedição de Juazeiro*; *Cenas e tipos*; *Varíola e vacinação no Ceará*; *Libertação do Ceará, seca de 1915* e *História da seca no Ceará (1877-1979)*. Neste último título, inaugurou sua escrita de caráter documental com o fim de registrar as duras consequências sofridas pelo povo, a partir das deliberações políticas dos governantes, que pareciam não ter nenhum compromisso com a mitigação das muitas dores enfrentadas pela população, sobretudo a mais pobre e, por isso, mais necessitada da ação governamental. Nesse livro, o escritor destaca que, em 1878, no auge de um triênio de secas que assolaram impiedosamente o Ceará, o senhor Silveira da Mota, que era senador pelo Rio Grande do Sul, visando reduzir a ajuda do governo federal ao Ceará, por motivações puramente políticas, já que os governantes locais, naquela situação, pertenciam a grupos opositores, alegara que o estado não passava por nenhuma seca, uma vez que continuava a exportar toneladas de farinha para outros estados. Indignado com a diminuição das verbas públicas enviadas e presenciando os muitos danos causados por essa ação, Teófilo procedeu a minucioso levantamento das certidões da alfândega para provar, por meio de seu livro, que durante aqueles anos de seca nenhum grão de farinha tinha saído do Ceará e que o senador Silveira da Mota havia falseado a verdade. Considerando que no presente artigo utilizamos como fonte de pesquisa, em grande medida, outro livro de Teófilo que segue a mesma linha contestatória e denunciativa, *Varíola e vacinação no Ceará* (1910), é oportuno destacar que a postura do intelectual de buscar delatar as ações políticas motivadas por interesses particulares, ainda que prejudiciais às populações de maior vulnerabilidade social, não se restringiu apenas a políticos específicos ou a seus desafetos pessoais, mas foi movida pela vontade de lançar luz sobre aquilo que considerava injusto, imoral e desumano na ação daqueles detentores de poder público, bem como de deixar para as gerações vindouras provas inequívocas de seu trabalho altruísta e abnegado como sanitarista por iniciativa pessoal, já que nunca fora investido em qualquer cargo político, anulando as injúrias que seus inimigos apregoavam, para, quem sabe, alcançar seu reconhecimento na posteridade (Soárez, 2009).

2 Oligarquia acciolyna refere-se ao período de 16 anos em que a cena política do Ceará esteve sob a influência do poderio da família de Antônio Pinto Nogueira Accioly (1840-1921), de 1896, ano em que foi eleito governador, a 1912, quando foi deposto por meio de grave insurreição popular. Nogueira Accioly, natural de Icó-CE, formado em ciências jurídicas na Academia de Olinda, já fortificava seu domínio público ao ocupar cargos de promotor na cidade de Icó e Saboeiro; juiz municipal de Fortaleza e Baturité; deputado geral (1880) e vice-presidente do Ceará (1884); senador (1889) e, finalmente, governador (1896). Seu governo, apesar de ter sido muito emblemático no tocante à modernização e embelezamento da cidade de Fortaleza, caracterizou-se, principalmente, pela postura truculenta e repressora aos seus adversários políticos, bem como pelo nepotismo e fraudes eleitorais, que contava com o apoio da política dos governadores, além de impopular política econômica, que sobretaxava comércio, indústrias e profissões liberais em 1904 e 1907. Diante dessas características, era crescente a insatisfação popular. Vale ressaltar que, em 1911, iniciou no Brasil, a partir da necessidade de o presidente Hermes da Fonseca conter forte oposição a ele nos estados, o “salvacionismo”, movimento encabeçado por setores militares e alguns segmentos da sociedade civil “salvacionismo”, que se baseou em intervenções militares nos estados, para destituir governadores e os substituir por nomeados pelo próprio presidente da República. O objetivo seria retirar do Estado brasileiro as oligarquias espalhadas por todo território nacional que ameaçavam o seu governo. Diante dessa nova conjuntura política federal, a oposição acciolyna ganhou força e lançou a candidatura do coronel Franco Rabelo, personalidade “salvacionista” para as eleições de 1912 ao governo estadual, contra a candidatura de Nogueira Accioly ou de qualquer de seus apadrinhados. A capital cearense conheceu ali as primeiras grandes passeatas de sua história. O ineditismo da iniciativa, envolvendo moças e crianças, acabou cristalizando e fortalecendo o sentimento de ruptura da sociedade de Fortaleza com Accioly, em apoio declarado a seu oponente. Particularmente na manifestação conhecida como Passeata das Crianças, em 21 de janeiro de 1912, a Polícia Militar investiu sobre os manifestantes com extrema violência, o que resultou na morte de uma criança. Esse foi o estopim para uma grande batalha por parte da população, que, por três dias, enfrentou a polícia, saqueou e depredou todos os símbolos que marcaram o governo, tais como os equipamentos modernizadores da cidade, bondes, postes de iluminação pública, praças, jardins etc. Diante da iminência de ter a sede do governo invadida pelos revoltosos, o oligarca, a fim de preservar a própria vida, renuncia ao governo do estado em 24 de janeiro de 1912 (Porto, 1993).

3 Em 19 de março de 1908, foi aprovado regulamento que rebatizava o Instituto de Patologia Experimental de Manguinhos, fundado em 25 de maio de 1900, como Instituto Oswaldo Cruz, em homenagem ao médico Oswaldo Cruz. A fundação foi inaugurada originalmente para fabricar soros e vacinas contra a peste bubônica. Pelas mãos do jovem bacteriologista Oswaldo Cruz, o instituto foi responsável pela reforma sanitária que erradicou a epidemia de peste bubônica e a febre amarela da cidade do Rio de Janeiro, então capital federal. A mudança vivenciada pela instituição em 1908 não se restringiu apenas à troca de nome, ela passava, também, a partir daquele regulamento, a ter “inteira e franca autonomia nas investigações científicas”, além disso, foi retirada do organograma da Diretoria Geral de Saúde Pública e atrelada diretamente ao Ministério da Justiça, o que colocava os direitos de ambas as instituições em pé de igualdade (Benchimol, 2020).

4 Vale destacar que as notícias citadas neste artigo foram retiradas do livro *Varíola e vacinação no Ceará*, publicado em 1910. Conforme sinalizamos no tópico introdutório deste texto, Rodolfo Teófilo reuniu vários documentos, entre os quais notícias publicadas por periódicos cearenses e de outros estados, referentes ao trabalho por ele desempenhado.

5 No isolamento horizontal, a orientação é para que todos permaneçam em isolamento, com o fim de limitar ao máximo o contato entre as pessoas para diminuir a proliferação do vírus. Apesar de causar impactos graves na economia, essa medida fazia-se essencial para evitar aumento descontrolado dos casos da doença e o colapso do sistema de saúde. Já no isolamento vertical, o qual foi defendido por Bolsonaro e por grande parte de seus apoiadores, apenas as pessoas do grupo de risco (idosos e pessoas com comorbidades) permaneceriam isoladas, sendo permitido aos jovens e indivíduos saudáveis circular normalmente. Todavia, durante o período mais crítico da pandemia de covid-19, sobretudo antes do desenvolvimento de uma vacina, a OMS foi contra essa forma de isolamento, uma vez que os jovens poderiam contrair e transmitir a doença aos mais vulneráveis, além de aumentar rapidamente o número de acometidos (Qual…, 1 jul. 2020).
